# c-JUN prevents methylation of p16INK4a (and Cdk6): the villain turned bodyguard

**DOI:** 10.18632/oncotarget.279

**Published:** 2011-05-28

**Authors:** Karoline Kollmann, Gerwin Heller, Veronika Sexl

**Affiliations:** ^1^ Institute of Pharmacology and Toxicology, University of Veterinary Medicine, Vienna, Austria; ^2^ Clinical Division of Oncology, Department of Medicine I, Comprehensive Cancer, Medical University of Vienna (MUV), Vienna, Austria

**Keywords:** AP-1, CDK6, p16, leukemia

## Abstract

A novel way by which the AP-1 factor c-JUN interferes with tumorigenesis has recently been elucidated [1]. In a model of murine leukemia, c-JUN prevents the epigenetic silencing of the cell cycle kinase CDK6. In the absence of c-JUN, CDK6 is down-regulated and the 5'region of the gene is methylated. Down-regulation of CDK6 results in significantly delayed leukemia formation. Here we show that c-JUN is also involved in protecting the promoter region of the tumor suppressor *p16^INK4a^,* which is consistently methylated over time in c-JUN deficient cells. In cells expressing c-JUN, *p16^INK4a^* promoter methylation is a less frequent event. Our study unravels a novel mechanism by which the AP-1 factor c-JUN acts as a “bodyguard”, and preventing methylation of a distinct set of genes after oncogenic transformation.

## THE AP-1 TRANSCRIPTION FACTOR FAMILY - TUMOR SUPPRESSORS AND PROMOTERS

AP-1 (Activator Protein-1) transcription factors are basic leucine-zipper (bZIP) proteins [2-5]. They act as dimeric transcription factors composed of members of the JUN family (c-JUN, JUNB, JUND), forming homo- or heterodimers with members of the FOS, ATF (activating transcription factor) and MAF (musculoaponeurotic fibrosarcoma) protein family [6-7]. The expression and physiological role of the diverse AP-1 complexes is cell type- and even differentiation state-dependent [8-10]. AP-1 complexes modulate transcription of target genes by binding to their TRE or CRE consensus elements [3],[11-13],[8, 12, 14]. Consequently AP-1 dimers are involved in the regulation of various biological processes such as proliferation, differentiation and apoptosis [3, 15]. It is therefore not surprising that AP-1 factors are involved in cellular transformation and tumorigenesis [3, 16-17].

c-JUN was first discovered as the homologue of the viral oncoprotein v-JUN, which induces avian sarcoma [18-19]. Early evidence revealed a cooperation of c-JUN with oncogenic RAS in cellular transformation [4, 20-21]. On the other hand, some AP-1 family members suppress and block tumor development [4, 22-23]. This task has been previously been attributed to JUNB, inactivation of which provokes the development of myeloproliferative disorders [24].

Despite the similarity in their primary structure and DNA binding specificity, c-JUN and JUNB differ in transcriptional capacity. Both may act either as a transcriptional activator or as a repressor depending on the promoter context and the heterodimerization partner [4, 25-31]. In some tissues c-JUN and JUNB even exert antagonistic functions in biological processes such as cell proliferation [4, 26-29, 31]. Thus AP-1 is able to modulate opposing functions, such as promoting or suppressing tumor development.

## C-JUN - ACCELERATING TUMORIGENESIS BY DRIVING TRANSCRIPTION

Since the initial discovery of v-JUN, solid evidence has accumulated linking AP-1 members and in particular c-JUN to tumor development. Using mouse models, c-JUN has been implicated in tumor formation in both skin and liver [32-36]. Inhibiting c-JUN activity in basal keratinocytes blocks chemically induced papilloma formation because of the lack of expression of the AP-1 target genes [37]. In hepatocellular carcinoma, gene deletion of *c-Jun* after tumor onset leads to a significant reduction in tumor size. This effect has been explained as resulting from increased apoptosis induced by c-JUN dependent suppression of the pro-apoptotic gene p53 [36, 38]. Upon loss of c-JUN, p53 mediated apoptosis kicks in and leads to a significant reduction of tumor burden [36]. Similarly, murine embryonic fibroblasts (MEFs) lacking c-JUN show severe cell cycle abnormalities with a block in the G1-phase of the cell cycle [39]. These fibroblasts demonstrate increased expression of the pro-apoptotic gene p53 and its target gene, the cell cycle inhibitor p21CIP [38]. As in hepatocytes, c-JUN negatively regulates the transcription of p53 in MEFs by direct binding to the promoter region of p53. The concomitant deletion of *p53* rescues the apoptotic and proliferative disadvantage [38, 40].

Besides c-JUN's “survival” function in interfering with p53 induced apoptosis, c-JUN has been shown to drive cell proliferation. One defined “hallmark” of tumor formation is a deregulated cell cycle and c-JUN's ability to effect this represents a further way in which the protein contributes to tumorigenesis. A direct pro-proliferative function of c-JUN is mediated through the regulation of CyclinD1 [38-39, 41]. c-JUN is able to bind to the CyclinD1 promoter and induces transcription in a phosphorylation-dependent manner, as the exchanges of critical serine residues to alanine (*Jun^AA/AA^*) decrease CyclinD1 transcription [30]. Both CyclinD1 and c-JUN are overexpressed in a broad range of human cancer and both molecules are considered proto-oncogenes [10, 42-45]. Another cell-cycle regulator under the control of AP-1 members is the cell cycle inhibitor p16^INK4a^. Several AP-1 binding sites within the *p16^INK4a^* promoter enable AP-1 to control expression of the gene. JUNB has been defined as a positive regulator and induces p16^INK4a^ expression [27, 46].

## A NOVEL MECHANISM FOR C-JUN

An additional cell cycle component regulated by c-JUN is the G1-cell cycle kinase CDK6. CDK6 is most closely related to CDK4 and is required to allow cells to progress through the G1-phase of the cell cycle after binding to D-type Cyclins [47-50]. CDK6 and CDK4 generally have overlapping and redundant functions but CDK6, unlike CDK4, has been recently recognized to be a regulator of differentiation [51-54].

When we analyzed pro B-cells transformed by the oncoprotein BCR-ABL we found a peculiar regulation of CDK6 downstream of c-JUN [1]. Over time BCR-ABL-transformed cells lacking c-JUN had lower levels of CDK6 protein as well as mRNA. The decline in protein expression was independent of c-JUN phosphorylation as CDK6 was stably expressed in BCR-ABL^+^ *Jun^AA/AA^* cells, in which the mutant JUN protein can no longer be phosphorylated. Interestingly, the reduction of CDK6 was accompanied by methylation of the 5'region of *Cdk6* and could be reversed by treatment of the cells with the DNA methyltransferase (DNMT) inhibitor 5-aza-2'-deoxycytidine (Aza-dC). Two AP-1 binding sites are found in the vicinity of the methylated region. This led us to suggest that c-JUN exerts a “bodyguard” function, being required to prevent the *Cdk6* promoter from being silenced. Only in the absence of c-JUN can methylation of the 5'region of *Cdk6* take place and CDK6 expression be silenced. This effect is restricted to transformed cells and induced by the oncogenic event, as primary non-transformed lymphoid cells display unaltered CDK6 expression levels. It is attractive to speculate that c-Jun may also shape age-related DNA methylation by acting as a “bodyguard” to protect distinct DNA regions during aging [55-57].

Altering CDK6 expression has major consequences for leukemia and lymphoma development. Using *Cdk6^-/-^* animals we detected a significant delay in tumor formation. Thus, it is safe to conclude that by preventing the silencing of CDK6 c-JUN accelerates and promotes leukemogenesis. In the same murine model of BCR-ABL^+^ leukemia, JUNB exerts an antagonistic role. In accordance with its role as tumor suppressor, the enforced expression of JUNB suppresses tumor formation and the lack of JUNB lead to a highly aggressive and rapidly progressing disease. Again we found a role for CDK6; the lack of JUNB was accompanied by high CDK6 expression levels. How precisely JUNB interferes with the regulation of CDK6 remains to be determined [46].

**Figure 1:**
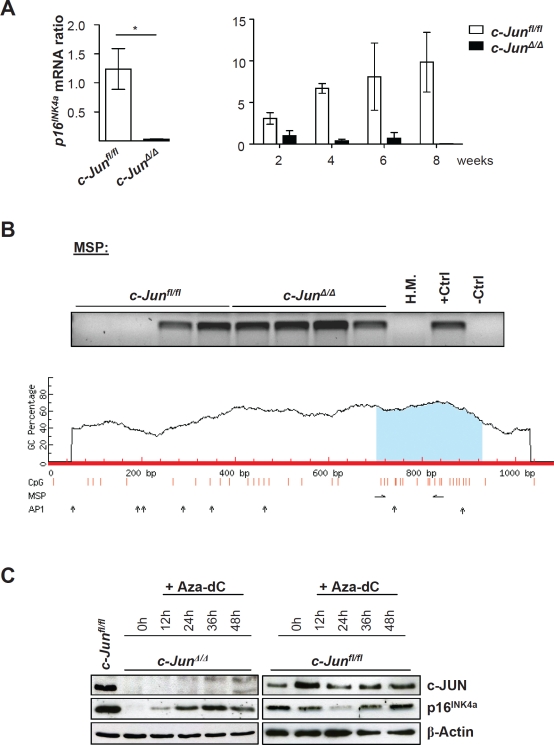
*p16^INK4a^* mRNA levels are down-regulated in *c-Jun^Δ/Δ^ p185^BCR-ABL^*-transformed cell lines A) *p16^INK4a^* mRNA levels of *c-Jun^Δ/Δ^* and *c-Jun^fl/fl^* cells 2, 4, 6 and 8 weeks after *p185^BCR-ABL^* transformation were analyzed by q-PCR. The fold change compared to *c-Jun^Δ/Δ^* 2 weeks *p16^INK4a^* mRNA level is shown. Results were normalized by comparison to their *Gapdh* mRNA expression. B) upper panel: Methylation-specific PCR analysis of *p16^INK4a^* in stable *c-Jun^fl/fl^ and c-Jun^Δ/Δ^* cell lines as detected by MSP analysis. A visible PCR product indicates the presence of methylated alleles. Abbreviations: *H.M.*, bone marrow of a healthy mouse; +Ctrl (control for methylated samples); -Ctrl (control for unmethylated samples). Lower panel: Graphical overview of the CpG island associated with *p16^INK4a^* (*Cdkn2a)* (ENSMUSG00000044303). The following genomic region is shown: NCBIM37:4:88927717:88928797:-1. Vertical bars (orange) indicate the location of CpG dinucleotides, horizontal arrows indicate MSP primer binding sites and vertical arrows indicate AP1 transcription factor binding sites predicted using the transcription factor binding profile database JASPAR (http://jaspar.genereg.net/). C) Immunoblot for c-JUN and p16INK4a of *c-Jun^Δ/Δ^* and *c-Jun^fl/fl^* cells after 12, 24, 36 and 48 hours of Aza-dC treatment. β-Actin served as loading control. One representative set of data is depicted.

CDK6 is not the only cell cycle regulator under the control of c-JUN. We found a consistent downregulation of the tumor suppressor and cell cycle inhibitor p16^INK4a^ in c-JUN deficient BCR-ABL+ cells [1]. The levels of both the protein and the mRNA (Figure 1A) of p16^INK4a^ decline with time after BCR-ABL-transformation, as verified by micro-array experiments [1].

**Scheme 1:**
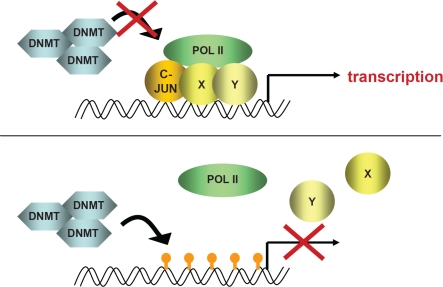
A transcriptional complex, including c-JUN, binds to DNA and methylation is inhibited In the absence of c-JUN, DNMTs can bind and methylate gene promoter region

As shown for *Cdk6*, we found promoter methylation of *p16^INK4a^* in all stable BCR-ABL+ *c-Jun^Δ/Δ^* cell lines. In the case of wild type, only half the cell lines displayed methylated CpG islands (Figure 1B). Treatment of the cells with Aza-dC reverted the reduction of p16^INK4a^ in cells lacking c-JUN (Figure 1C). Interestingly, no changes could be induced by treating wild type cells with this agent, indicating that additional regulatory mechanisms had shut off p16^INK4a^ in the tumor cell lines. As is the case for *Cdk6*, the promoter region of the *p16^INK4a^* gene contains a number of AP1-binding sites, which is consistent with our model, that c-JUN is required to protect the *p16^INK4a^* promoter from being silenced by methylation.

## CONCLUSION

c-JUN is a common regulator of cell cycle components that has been described to have a direct role in regulating the transcription of p53 and CyclinD1. Based on our studies we postulate a novel mechanism for how c-JUN accelerates leukemogenesis and regulates genes required for cell cycle progression in tumor cells. We propose that binding of c-JUN to the promoter region exerts a protective function and prevents methylation and silencing of the genes. This function of c-JUN has recently been demonstrated for the cell cycle kinase CDK6. In the absence of c-JUN, CDK6 is downregulated accompanied by methylation of the 5'region. We now show that the same mechanism occurs at the promoter region of the cell cycle inhibitor and tumor suppressor *p16^INK4a^*. Our results indicate a novel mechanism by which AP-1 factors modulate tumor formation.
